# Cost-Effectiveness Analysis of Pembrolizumab Plus Pemetrexed and Platinum *Versus* Chemotherapy Alone as First-Line Treatment in Metastatic Non-Squamous Non–Small Cell Lung Cancer: A Reconstruction of Partitioned Survival Model Based on Time Dependent Pricing Mechanism of Patient Assistance Program

**DOI:** 10.3389/fonc.2021.768035

**Published:** 2021-11-26

**Authors:** Yuanyi Cai, Wen Hui, Min Zhu, Mingyue Zhang, Zhixiang Gao, Huazhang Wu

**Affiliations:** ^1^ Department of Health Service Management, School of Health Management, China Medical University, Shenyang, China; ^2^ West China Hospital, Sichuan University, Chengdu, China; ^3^ Department of Health Economics, School of Health Management, China Medical University, Shenyang, China; ^4^ Department of Pharmacy, Affiliated Central Hospital of Shenyang Medical College, Shenyang, China

**Keywords:** non-small cell lung cancer, pembrolizumab, cost-effectiveness analysis, partitioned survival model, patient assistance program

## Abstract

**Objectives:**

A new patient assistance program (PAP) for pembrolizumab was started in China in 2021. The researchers aimed to evaluate the economic outcomes of pembrolizumab plus pemetrexed and platinum *versus* chemotherapy alone in the first-line treatment of patients with metastatic non-squamous non-small cell lung cancer, based on the pricing mechanism of PAP.

**Material and Methods:**

Survival analysis and partitioned survival model were performed to evaluate the incremental cost-effectiveness ratio (ICER) in the pembrolizumab group compared with the chemotherapy group. Survival probabilities were extracted from the data of the KEYNOTE-189 trial. Cost and utility data were gathered from published literature. The pricing mechanism of PAP was set in each cycle in the partitioned survival model, according to the progression-free survival (PFS) data of the KEYNOTE-189 trial, which included PFS-1 and PFS-2. Deterministic sensitivity analysis and probabilistic sensitivity analysis were conducted.

**Results:**

The ICER of the pembrolizumab group *versus* chemotherapy group was $65,272/quality-adjusted life year (QALY), which still exceeded the willingness-to-pay threshold of three times per capita gross domestic product (GDP) of China ($33,581.22), although PAP was calculated. Sensitivity analysis implied that the price of chemotherapeutic drugs combined with pembrolizumab was one of the main influencing factors of ICER.

**Conclusions:**

Due to various prices set by PAP and the payment for combined chemotherapy, the economic advantage of pembrolizumab plus chemotherapy in the first-line treatment of non-small cell lung cancer (NSCLC) is still not achieved in China.

## 1 Introduction

Lung cancer is currently one of the most malignant tumors with the highest incidence and mortality worldwide. In 2018, the number of new cases of lung cancer was 2.094 million, accounting for 11.58% of new cases of tumors. The number of deaths due to lung cancer was 1.761 million, accounting for 18.43% of global tumor deaths (1). In China, there were 0.787 million new cases of lung cancer in 2015 with 0.52 million males and 0.267 million females, and 0.631 million deaths, based on the newly updated data of National Cancer Center in 2019 (2).

Lung cancer includes small-cell lung cancer (SCLC) and non-small cell lung cancer. Majority of lung cancers are non-small cell lung cancer, which accounts for approximately 85%–90% of all lung cancers (3). In recent years, the emergence of immunotherapy has given hope of longer survival to advanced patients, and has also increased the possibility of cure inpatients at an early stage. For non-small cell lung cancer (NSCLC), PD-1 and PD-L1 inhibitors are mainly used in clinical practice, which block the interaction of PD-1 and PD-L1, activate the immune system, and prevent tumor immune escape. In China, pembrolizumab, a representative PD-1 inhibitor, is gradually being approved for more indications. On April 2, 2019, the National Medical Products Administration (NMPA) of the People’s Republic of China approved pembrolizumab combined with pemetrexed and cisplatin chemotherapy as first-line treatment of metastatic non-squamous NSCLC, without sensitizing EGFR or ALK mutations. On September 30, 2019, NMPA approved pembrolizumab as a single agent for the first-line treatment of locally advanced or metastatic NSCLC with PD-L1 TPS ≥ 1%, without sensitizing EGFR or ALK mutations. On November 25, 2019, NMPA approved pembrolizumab combined with paclitaxel or paclitaxel (albumin-bound) and carboplatin chemotherapy for the first-line treatment of metastatic squamous NSCLC (4). With the approval of more indications of pembrolizumab, an increasing number of patients are receiving pembrolizumab treatment. Although patients’ lives are prolonged, the expensive price of immunotherapy also places a heavy economic burden. The pharmacoeconomic analysis of pembrolizumab and chemotherapy first-line combination treatment in Chinese patients conducted by Wan and Jiang showed that the incremental cost-effectiveness ratio (ICER) values were $92,533/quality-adjusted life-year (QALY) and $96,644/QALY, respectively (5, 6), which exceeded three times the per capita gross domestic product (GDP) of $33,581.22 in China in 2020 (7).

In December 2020, the National Healthcare Security Administration and the Ministry of Human Resources and Social Security of the People’s Republic of China started a new round of price negotiations on the access to innovative medicines. Although pembrolizumab sustained its original price, a new patient assistance program (PAP) started under the circumstance of fierce price competition from other PD-1 inhibitors. The purpose of the present study was to explore the construction of a pharmacoeconomic model, considering the cost parameters influenced by PAP, and evaluate the change in ICER for patients.

## 2 Material and Methods

### 2.1 Patients and Clinical Treatments

This study was based on a global, double-blind, placebo-controlled phase 3 clinical trial (KEYNOTE-189). The trial aimed to compare the combination of pemetrexed and a platinum-based drug plus either pembrolizumab or placebo as first-line treatment in patients with non-squamous NSCLC (8).

### 2.2 Model Structure

#### 2.2.1 Survival Analysis

**Figure 1:**
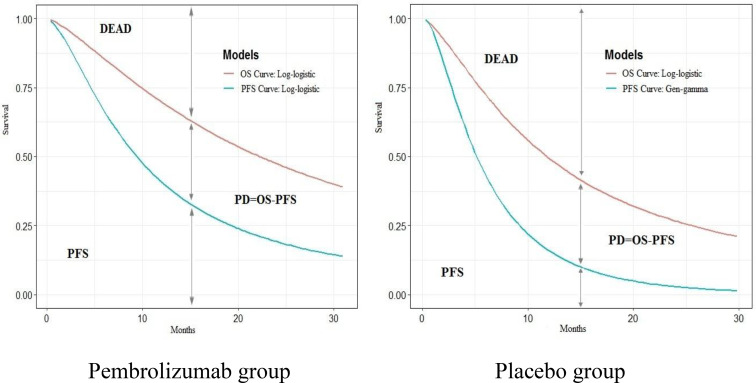
Construction of partitioned survival model in the pembrolizumab and placebo group.

WebPlotDigitizer (https://apps.automeris.io/wpd/index.zh_CN.html) was used to extract the data of the overall survival (OS) and progression-free survival (PFS) curves in the KEYNOTE-189 trial. Based on the OS and PFS data, the survHE package of R (V4.0.3) was used to fit and extrapolate the PFS and OS curves. According to the Akaike information criterion (AIC) and Bayesian information criterion (BIC), combined with visual inspection, log-logistic distribution for OS curve (shape=1.351,7, scale=22.160,0, AIC=1,925.219, BIC=1,933.251) and PFS curve (shape=1.532,8, scale=9.378,1, AIC=2,248.140, BIC=2,256.173) in the pembrolizumab group; log-logistic distribution for OS curve (shape=1.422,9, scale=11.754,6, AIC=1,142.539, BIC=1,149.194), and the gengamma distribution for PFS curve (mu=1.738,0, sigma=0.875,6, Q=0.325,3, AIC= 1,088.212, BIC=1,098.196) in the placebo group were selected (**Figure 1**) (see **Supplementary Material**) (9).

#### 2.2.2 Model Construction

**Figure 2:**
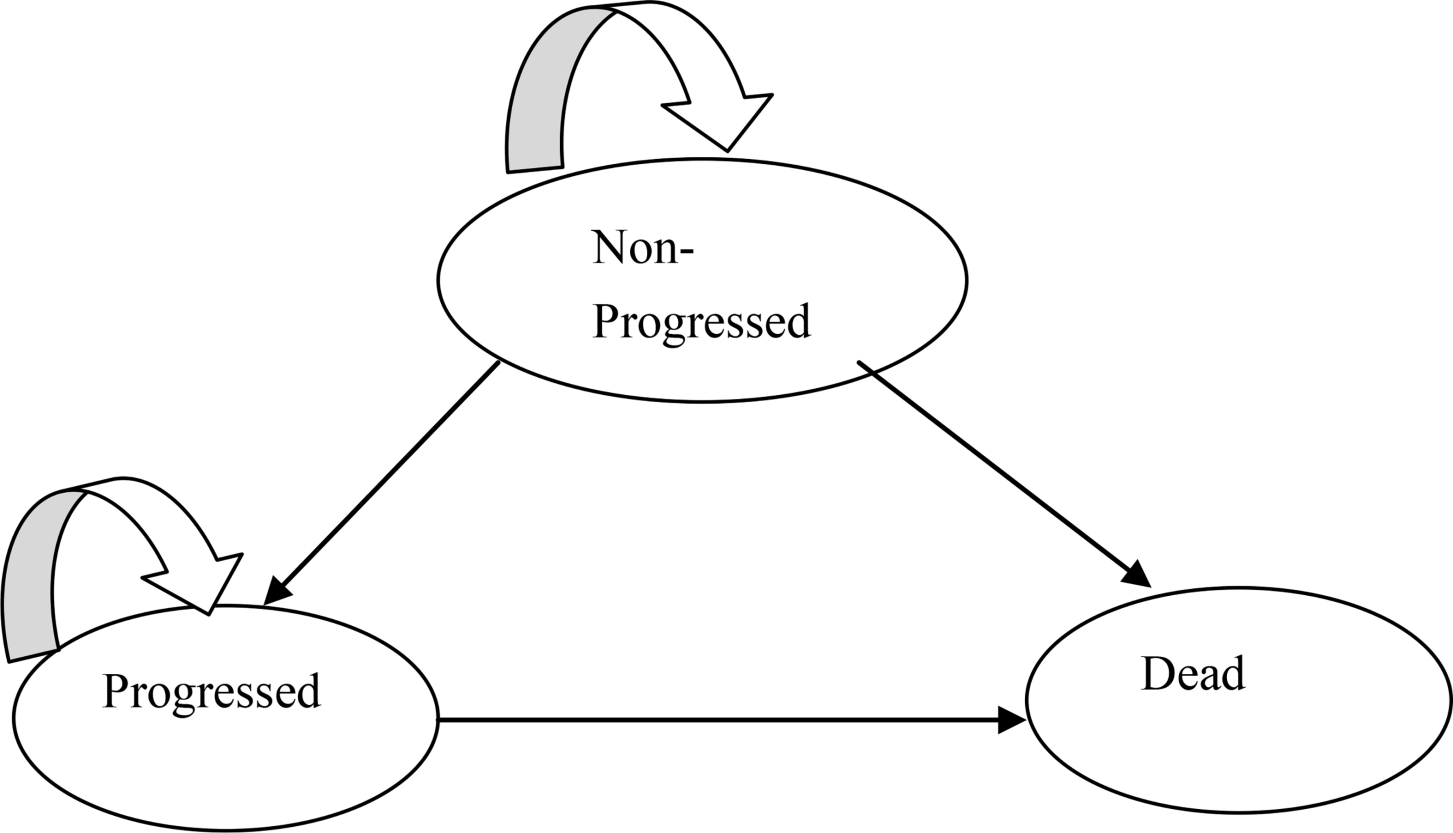
Three main health states of partitioned survival model.

The Heemod package of R was used to construct the partitioned survival model. Three states were included: progression-free survival, progressive disease (PD), and death (**Figure 2**). Each cycle was set to 3 weeks in this model, according to the medication plan of the KEYNOTE-189 trial. A total of 10 years was simulated, which nearly reached to a lifetime, considering the poor prognosis of patients with metastatic NSCLC. The main outcomes were cost and QALYs, which were discounted at a rate of 5% (10), and three times per capita GDP of 2020 in China was selected as the threshold for ICER. Meanwhile, cost and health outcome data were subjected to half-cycle correction.

### 2.3 Treatment Costs

This analysis adopted a health care perspective in China, which only analyzed direct medical costs. It included the cost of the drug, management of serious adverse events, medical services, and follow-up. The drug prices were derived from the median value of winning bid prices in Chinese provinces, and the cost of medical service and follow-up were derived from the median value of cost in tertiary medical institutions in sample areas (11), and other costs were derived from published literature (**Table 1**).

**Table 1:** Parameters’ value in the partitioned survival model.

	Base Value ($)	DSA range ($)	PSA Distribution	Reference
Price of pembrolizumab	2,768.50/100mg	2,076.45-3,460.70	lognormal	(11)
Price of carboplatin	0.17/mg	0.13-0.22	lognormal	(11)
Price of pemetrexed	330.96/100mg	248.30-413.78	lognormal	(11)
Price of nivolumab	1,430.76/100mg	1,073.07-1,788.45	lognormal	(12)
Price of docetaxel	200.86/200mg	150.65-251.08	lognormal	(13)
Cost of medical service of pembrolizumab therapy per cycle	8.69	6.52-10.86	lognormal	(11)
Cost of medical service of chemotherapy per cycle	48.71	36.53-60.89	lognormal	(11)
Cost of imaging examination and laboratory test per cycle	62.02	46.52-77.53	lognormal	(4, 11)
Utility of PFS	0.804	0.683-0.925	beta	(14)
Utility of PD	0.321	0.273-0.369	beta	(14)
Utility of death	0	–	–	–
Discount rate	5%	0-8%	–	(10)

DSA, deterministic sensitivity analyses; PSA, probabilistic sensitivity analyses.

All the costs were converted into United States dollars using the exchange rate: 1 US dollar = 6.4721 Chinese yuan renminbi.

#### 2.3.1 Drug Cost

Two phases of the medication plans were involved on the KEYNOTE-189 trial (see **Supplementary Material**). Thus, time-dependent cost parameters were considered in the R program. In the first phase, patients were assigned to receive 200 mg pembrolizumab plus pemetrexed (870mg) and carboplatin (511.87mg) every 3 weeks for 4 cycles, followed by pembrolizumab and pemetrexed for up to 35 cycles, followed by pemetrexed maintenance therapy until disease progression in the pembrolizumab group. The original pembrolizumab price was $2,768.50/100mg (11). Patients may participate in the patient assistance program in China. According to the newest PAP (program name: KEY TO LIFE) of pembrolizumab in 2021, patients who bought the first two cycles of pembrolizumab would receive the next two cycles for free, continuing to buy the fifth and sixth cycles of pembrolizumab; next cycles remaining for up to 2 years (35 cycles) would also be free. Considering PAP, the price of pembrolizumab was different due to patient survival time. If pembrolizumab was more effective for patients, the price would be lower in each cycle. In the KEYNOTE-189 trial, the median PFS was 9.0months, which was equivalent to 12 cycles. After PAP, the average price of patients with PFS in the first cycle was set as follows:

$\text{Cos}{\text{t}}_{12-\text{original}}=\;{\text{P}}_{\text{original}}\times 2\times 12$ (1)

$\text{Cos}{\text{t}}_{12-\text{PAP}}=\;{\text{P}}_{\text{original}}\times 2\times 4$ (2)

${\text{r}}_{1}=\text{ Cos}{\text{t}}_{12-\text{PAP}}/\text{Cos}{\text{t}}_{12-\text{original}}$ (3)

${\text{P}}_{12-\text{PAP}}=\;{\text{P}}_{\text{original}}\times {\text{r}}_{1}$ (4)

${\text{P}}_{1-\text{average}}=\;{\text{P}}_{12-\text{PAP}}$ (5)

If the patient used pembrolizumab for two years, which was equivalent to 35 cycles. After PAP, the average price of patients with PFS in the 35th cycle was set as follows:

$\text{Cos}{\text{t}}_{35-\text{original}}=\;{\text{P}}_{\text{original}}\times 2\times 35$ (6)

$\text{Cos}{\text{t}}_{35-\text{PAP}}=\;{\text{P}}_{\text{original}}\times 2\times 4$ (7)

${\text{r}}_{2}=\text{ Cos}{\text{t}}_{35-\text{PAP}}/\text{Cos}{\text{t}}_{35-\text{original}}$ (8)

${\text{P}}_{35-\text{PAP}}=\;{\text{P}}_{\text{original}}\times {\text{r}}_{2}$ (9)

${\text{P}}_{35-\text{average}}=\;{\text{P}}_{35-\text{PAP}}$ (10)

Thus, during the 35 cycles, the price of pembrolizumab in each cycle was time-dependent and was set as follows:

${\text{P}}_{\text{i cycle}}={\text{P}}_{1-\text{average}}-[({\text{P}}_{1-\text{average}}-{\text{P}}_{35-\text{average}})/34]*\left(\text{Marcov }\_\text{cycle }-1\right)\left(\text{i }=\;1\text{ to }35\right)$ (11)

Markov_cycle was internal cycle counter of R language.

Patients were assigned to receive saline placebo plus pemetrexed and carboplatin for 4 cycles, followed by saline placebo and pemetrexed maintenance therapy until disease progression in the placebo group.

After disease progression, 44.6% of patients received ≥1 subsequent therapy in the pembrolizumab group. Among these patients, 13.4% received ≥1 subsequent PD-1 or PD-L1 inhibitor, according to the KEYNOTE-189 trial. Based on this information, 13.4% of patients were assumed to choose nivolumab (185.58mg every 2 weeks), and 31.2% of patients were assumed to choose docetaxel (104.4mg every 3 weeks) (13). Meanwhile, in the placebo group, 59.2% of patients received ≥1 subsequent therapy. Among these patients, 53.9% received ≥1 subsequent PD-1 or PD-L1 inhibitors. In patients receiving PD-1 or PD-L1 inhibitors, 40.8% of the patients were crossed over to the trial group. Thus, 5.3% of patients were assumed to choose docetaxel, 13.1% were assumed to choose nivolumab, and 40.8% patients were crossed over to use pembrolizumab in this study. PFS-2, which was defined as “the time from randomization to objective tumor progression on next-line treatment or death from any cause” in the KEYNOTE-189 trial, showed that the median PFS was 4.9 months in the placebo group. Thus, after about 4.1 months, patients underwent another progression (8), and the lower limiting value - 2.9 months, about 4 cycles were included in the analysis. Considering PAP, the average price of patients in the first cycle from PFS-1 to PFS-2 in the placebo group was set as follows:

$\text{Cos}{\text{t}}_{4-\text{original}}=\;{\text{P}}_{\text{original}}\times 2\times 4$ (12)

$\text{Cos}{\text{t}}_{4-\text{PAP}}=\;{\text{P}}_{\text{original}}\times 2\times 2$ (13)

${\text{r}}_{3}=\text{ Cos}{\text{t}}_{4-\text{PAP}}/\text{Cos}{\text{t}}_{4-\text{original}}$ (14)

${\text{P}}_{4-\text{PAP}}=\;{\text{P}}_{\text{original}}\times {\text{r}}_{3}$ (15)

$\text{P}{'}_{1-\text{average}}=\;{\text{P}}_{4-\text{PAP}}$ (16)

Due to the short period from PFS-1 to PFS-2 in the placebo group, the price of pembrolizumab in the following cycles was assumed to maintain the price of the first cycle.

${\text{P}}_{\text{j cycle}}=\text{P}{'}_{1-\text{average}}$ (17)

#### 2.3.2 Adverse Event Cost

Adverse events that were ≥3 grade and occurred in ≥3% of patients were analyzed, including nausea, anemia, fatigue, diarrhea, neutropenia, vomiting, dyspnea, asthenia, and thrombocytopenia. These adverse events were assumed to occur in the first cycle, because oncologists might change the anticancer drug if ≥3 grade adverse events frequently occur (11). Thus, adverse events were summed to the total cost only once in this study (**Table 2**).

**Table 2:** Adverse events and costs in the pembrolizumab group and chemotherapy group.

	Medical cost ($)	Pembrolizumab group Grade 3-5 (%)	Placebo group Grade 3-5 (%)	Reference
Nausea	14.62	3.5	4	(15)
Anemia	604.03	18.3	15.8	(16)
Fatigue	135.51	6.9	3.5	(15)
Diarrhea	5.90	5.2	3	(16)
Neutropenia	541.85	16	12.4	(15)
Vomiting	14.62	4	3	(15)
Dyspnea	343.18	4.2	5	(17)
Asthenia	135.51	6.7	3.5	(15)
Thrombocytopenia	550.91	8.4	6.9	(16)

#### 2.3.3 Medical Service and Follow-Up Cost

The costs of medical services included diagnostic fees, intravenous injection fees, nursing fees, and hospitalization fees. Follow-up items included CT, urine tests, blood tests, and blood biochemistry (11).

### 2.4 Utility

The utility of PFS was set to 0.804, and the utility of PD was set to 0.321, according to a health utility study in Chinese NSCLC patients by Nafees (14).

### 2.5 Sensitivity Analyses

To evaluate the stability of the results, deterministic sensitivity analyses and probabilistic sensitivity analyses were performed. DSA was conducted for all the parameters. A 25% change range was assumed for the parameters of drug price, medical service cost, and follow-up cost (18). A 15% change range was assumed for the parameters of PFS and PD utility (11), and a 0%–8% was assumed for the discount rate (10). In PSA, a Monte Carlo simulation of 10,000 iterations was performed for more stable result. The log-normal distribution was selected for the cost parameters, and the beta distribution was selected for utility parameters (19).

## 3 Results

### 3.1 Base-Case Results

In the base-case analysis, the average cost-effectiveness ratios (ACER) of patients in the pembrolizumab group and placebo group were $71,494.32/QALY, $76,734.17/QALY, respectively. Patients in the pembrolizumab group obtained an additional 0.64 QALYs, but needed to pay an extra $41,774.08. The incremental cost-effectiveness ratio was $65,272/QALY. The value of GDP per capita was $11,193.74 in China in 2020 (7). Even though PAP was considered, the ICER remained more than the threshold of three times the GDP per capita ($33,581.22).Considering net benefit, the net monetary benefits (NMB) of patients in the pembrolizumab group and placebo group were $ -53,078.3 and $ -32,796.2, respectively (**Table 3**).

**Table 3:** Cost-effectiveness Analysis of pembrolizumab group *versus* placebo group.

	Cost ($)	Incremental Cost ($)	QALYs	Incremental QALYs	ICER ($/QALY)
Placebo group	58,317.97		0.76		
Pembrolizumab group	100,092.05	41,774.08	1.40	0.64	65,272

### 3.2 Sensitivity Analyses

**Figure 3:**
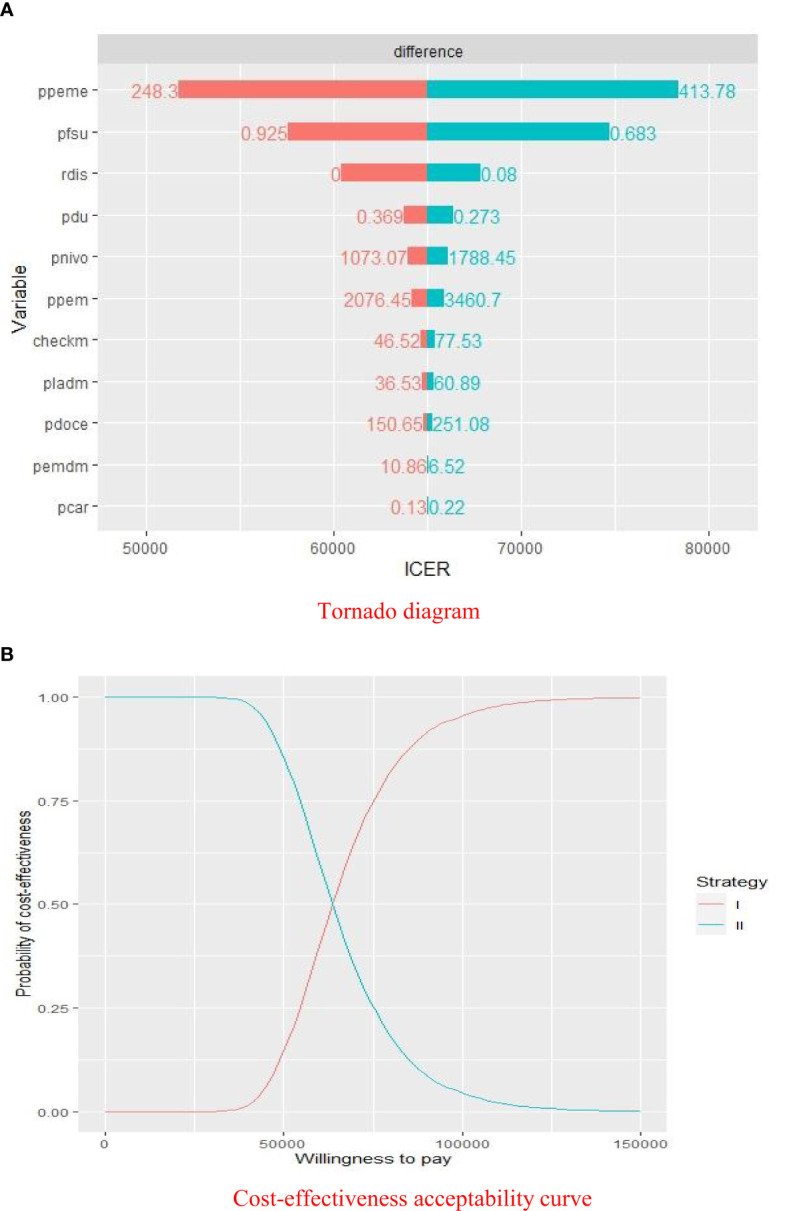
Sensitivity analyses of pembrolizumab group *versus* placebo group. **(A)** Deterministic sensitivity analysis of two groups. **(B)** Probabilistic sensitivity analysis of two groups. ICER, $/QALY; ppeme, price of pemetrexed; pfsu, utility of PFS; rdis, discount rate; pdu, utility of PD; pnivo, price of nivolumab; ppem, price of pembrolizumab; checkm, cost of imaging examination and laboratory test per cycle; pladm, cost of medical service of chemotherapy per cycle; pdoce, price of docetaxel; pemdm, cost of medical service of pembrolizumab therapy per cycle; pcar, price of carboplatin; Willingness to pay, $/QALY; strategy I, pembrolizumab plus pemetrexed and platinum; strategy II, chemotherapy alone.

The results of the deterministic sensitivity analyses were presented in a Tornado diagram, which indicated that the price of pemetrexed, utility of PFS, and discount rate were the main influencing factors of ICER. Cost-effectiveness acceptability curve was generated to show the probabilities of cost-effectiveness, which revealed that the pembrolizumab strategy was not cost-effective compared with the placebo strategy at the threshold of $33,581.22/QALY (**Figure 3**).

## 4 Discussion

In December 2020, a new round of adjustment of the National Basic Medical Care Insurance Medicine Catalog was carried out in China. Pharmacoeconomics was applied to the price negotiation of innovative medicines during the adjustment of the medical insurance catalog. In the negotiation process, main factors such as adverse events, international prices, and competitiveness were considered. Finally, three types of PD-1 inhibitors, including Tislelizumab, Toripalimab, and camrelizumab, entered the new medical insurance catalog through price cuts. The prices of these three PD-1 inhibitors were reduced to $336.83 (100mg), $324.62 (240mg), and $452.40 (200mg), respectively, lower than 30% of their original price (20). The price-cut strategy was not used for pembrolizumab to maintain the global pricing system, which resulted in the failure to enter the medical insurance catalog. Instead, KEY TO LIFE, a competitive patient assistance program, started in 2021, which reduced the patients’ payments to some extent. However, the new PAP could not guarantee every patient to obtain equal price concessions, which led to the persistence of the economic burden. Our results revealed that the ICER was $65,272/QALY, which was significantly lower than the value reported by Wan and Jiang (5, 6), but still exceeded three times per capita GDP.

To the researchers’ knowledge, this study was the first to discuss the pricing mechanism of PAP in a partitioned survival model of NSCLC. If patients obtained a better treatment effect, the price of pembrolizumab would be much lower. In this study, according to the PFS-1 and PFS-2 in the KEYNOTE-189 trial, the pricing mechanism of PAP was coded in the R language, and a time-dependent pricing system for pembrolizumab in the partitioned survival model was established, which made the cost in each cycle more precise.

Although transnational pharmaceutical companies achieved a delicate balance between maintaining global pricing strategies and a competitive price in China through PAP, the pricing mechanism of PAP sets a threshold cost for patients, which would affect the risk averters’ initial choice of pembrolizumab. An alternative incentive method should be canceling the threshold cost (the first four cycles), and let patients pay the “effective part”. This new strategy would enable the company to obtain a balance between public welfare and profit, which also depends on the exploration and discovery of more precise prediction markers for treatment efficacy. A new PAP should be established for the following cycles to reduce the economic burden. In addition, installment or return of medical expenses to patients owing to ineffective treatment are other strategies employed for pharmaceutical companies, which were already adopted such as GlaxoSmithKline’ Strimvelis and Spark Therapeutics’ Luxturna.

In the long run, price cuts remain an important pathway to gain more market share from developing countries, especially China, which could reduce the transaction costs for transnational pharmaceutical companies, such as finding transaction partners (hospital, etc.), promoting, establishing contract relationships, and fulfilling contracts. Meanwhile, this could also be a trend to occupy the market through a price cut strategy, with the emergence of an increasing number of competitors in the global market, and increasing pressure on medical insurance payment control from an increasing number of countries.

Deterministic sensitivity analyses showed that the prices of other chemotherapeutic drugs used in combination with pembrolizumab were the important influencing factors of ICER, which suggested the possibility of further reducing the economic burden on patients. In addition, with the success of the KEYNOTE-042 trial, pembrolizumab as first-line monotherapy has been an important choice for patients with NSCLC (21). Recommendations have been made in the latest “Clinical practice guideline for stage IV primary lung cancer in China (2021 version)”: pembrolizumab plus platinum-containing chemotherapy is recommended; pembrolizumab as the monotherapy can also be chosen for patients with PD-L1 scores of 1-49% (4).”However, in the latest 2021 ASCO Annual Meeting, a research by Akinboro showed that patients with PD-L1 scores 1-49% receiving chemotherapy and immunotherapy had longer PFS and OS compared to patients treated with immunotherapy alone, with median PFS 7.7 *vs* 4.2 months and median OS 21.4 *vs* 14.5 months (22). Thus, for oncologists, decision making between monotherapy and combination therapy requires further supportive evidence from clinical trials.

This study has two limitations. First, in the KEYNOTE-189 trial, the patient’s overall survival time and progression-free survival time were the results of following the trial protocol. Therefore, the trial design was followed as much as possible in the analysis of cost in our study, especially at the time of disease progression, which might be different from oncologists’ real clinical decisions. Second, since pembrolizumab has been approved in the Chinese market for a short time, there remains a lack of evidence from a real-world study to further verify the long-term cost and effectiveness of pembrolizumab in patients in this study. Recently, a retrospective cohort study of nearly 20,000 medical insurance patients found that the survival benefit of using immune checkpoint inhibitors in the first-line treatment of NSCLC in the real world was much lower than that of clinical trials (23).

## 5 Conclusions

Considering the background of fierce competition in the Chinese immunotherapy market, a new PAP of pembrolizumab started in 2021, which reduced patients’ economic burden. However, due to various prices set by PAP and the payment allocated for combined chemotherapy, the economic advantage of pembrolizumab plus chemotherapy in the first-line treatment of NSCLC was not achieved.

## Data Availability Statement

The original contributions presented in the study are included in the article/**Supplementary Material**. Further inquiries can be directed to the corresponding author.

## Author Contributions

YC and HW studied the concept and made the whole design. YC, MZ, and MYZ collected the data from the literature. ZG gave the specific explanation about the use of anticancer drug. YC and WH established the model and conducted the analysis of data. YC were the main drafter of the manuscript. All authors contributed to the article and approved the submitted version.

## Funding

The Educational Department of Liaoning Province Funding Project (No. QNRW2020007). The funding source had no role in the preparation of this manuscript.

## Conflict of Interest

The authors declare that the research was conducted in the absence of any commercial or financial relationships that could be construed as a potential conflict of interest.

## Publisher’s Note

All claims expressed in this article are solely those of the authors and do not necessarily represent those of their affiliated organizations, or those of the publisher, the editors and the reviewers. Any product that may be evaluated in this article, or claim that may be made by its manufacturer, is not guaranteed or endorsed by the publisher.

## Supplementary Material

The Supplementary Material for this article can be found online at: https://www.frontiersin.org/articles/10.3389/fonc.2021.768035 /full#supplementary-material

## Abbreviations

PAP, Patient assistance program; ICER, Incremental cost-effectiveness ratio; PFS, Progression-free survival; QALY, Quality-adjusted life year; GDP, Gross domestic product; NSCLC, Non-small-cell lung cancer; SCLC, Small-cell lung cancer; NMPA, National Medical Products Administration; OS, Overall-survival; AIC, Akaike information criterion; BIC, Bayesian information criterion; PD, Progressive disease; DSA, Deterministic sensitivity analyses; PSA, Probabilistic sensitivity analyses; ACER, Average cost-effectiveness ratio; NMB, Net monetary benefit.
