# Supplementary material for: Cost-Effectiveness Analysis of Pembrolizumab Plus Pemetrexed and Platinum Versus Chemotherapy Alone as First-Line Treatment in Metastatic Non-Squamous Non–Small Cell Lung Cancer: A Reconstruction of Partitioned Survival Model Based on Time Dependent Pricing Mechanism of Patient Assistance Program
Source: Front Oncol. 2021 Nov 26;11:768035. doi: 10.3389/fonc.2021.768035 (PMC8661036; doi:10.3389/fonc.2021.768035)
Supplement: Supplementary file 1 [file DataSheet_1.docx]

**Supplementary material**

**1. Data Extraction**

WebPlotDigitizer was used to extract data from the OS and PFS curves in the KEYNOTE-189 trial, and packages of survHE, survival, and survminer in R language were used to realize the reconstruction (Figure S1-S4).The median OS and median PFS, based on original data from the KEYNOTE-189 trial, were compared with the reconstructive values (Table S1).


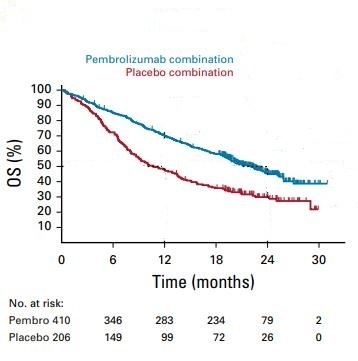

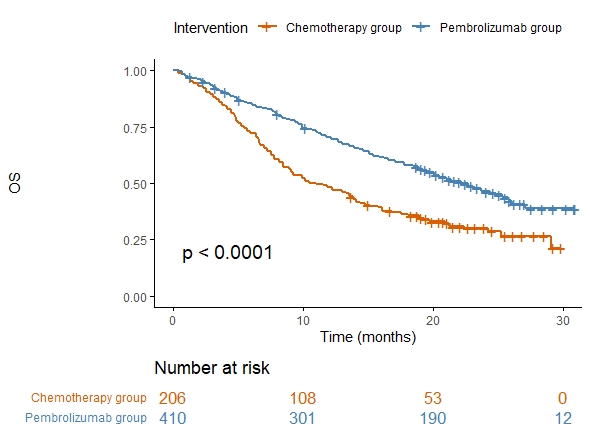


Figure S2. Reconstructive OS curve

Figure S1. Original OS curve


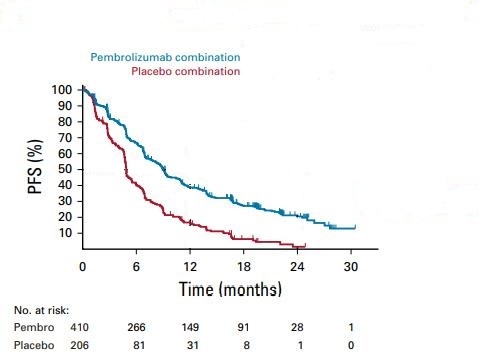

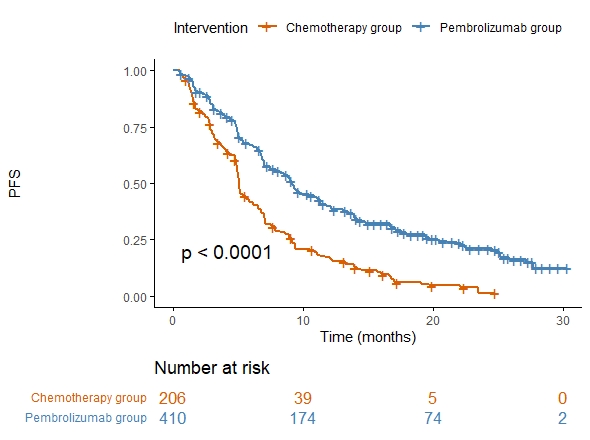


Figure S4. Reconstructive PFS curve

Figure S3. Original PFS curve

Table S1. Median OS and Median PFS of Original Trial and Reconstructive Model

| Group | Number at risk | Median OS/ Median PFS（months） | 95%CI（months） |
| --- | --- | --- | --- |
| OS curve |  |  |  |
| Trial OS in the pembrolizumab group | 410 | 22.0 | （19.5,25.2） |
| Model OS in the pembrolizumab group | 410 | 22.2 | （20.1,25.3） |
| Trial OS in the chemotherapy group | 206 | 10.7 | （8.7,13.6） |
| Model OS in the chemotherapy group | 206 | 10.7 | （8.9，14.1） |
| PFS curve |  |  |  |
| Trial PFS in the pembrolizumab group | 410 | 9.0 | （8.1,9.9） |
| Model PFS in the pembrolizumab group | 410 | 9.13 | （8.23,10.43） |
| Trial PFS in the chemotherapy group | 206 | 4.9 | （4.7,5.5） |
| Model PFS in the chemotherapy group | 206 | 5.04 | （4.88,5.85） |

**2. Fitting and extrapolation of PFS and OS curves**

According to Bayesian information criteria (BIC) and Akaike information criteria (AIC), visual inspection and statistical goodness-of-fit were conducted to determine the best fitting and extrapolation models for PFS and OS curves in the KEYNOTE-189 trial from seven main survival functions including exponential, gamma, gengamma, Gompertz, Weibull, loglogistic, and lognormal. In this part, PFS curves in the pembrolizumab and placebo groups were taken as examples.

**2.1 PFS curve in the pembrolizumab group**

Among the seven distributions, loglogistic, lognormal, and gengamma had the highest statistical fit, considering the values of AIC and BIC (Table S2). The three distributions were also more consistent with the trend of PFS curve in the pembrolizumab group, based on the KEYNOTE-189 trial, according to the visual inspection (Figure S5-S6). Finally, the loglogistic distribution with the best statistical fit was selected.

Table S2. PFS Curve in the Pembrolizumab Group

|  | exponential | gamma | gengamma | gompertz | weibull | loglogistic | lognormal |
| --- | --- | --- | --- | --- | --- | --- | --- |
| AIC | 2,268.041 | 2,261.232 | 2,249.122 | 2,270.035 | 2,264.765 | 2,248.140 | 2,248.315 |
| BIC | 2,272.057 | 2,269.265 | 2,261.170 | 2,278.067 | 2,272.797 | 2,256.173 | 2,256.347 |


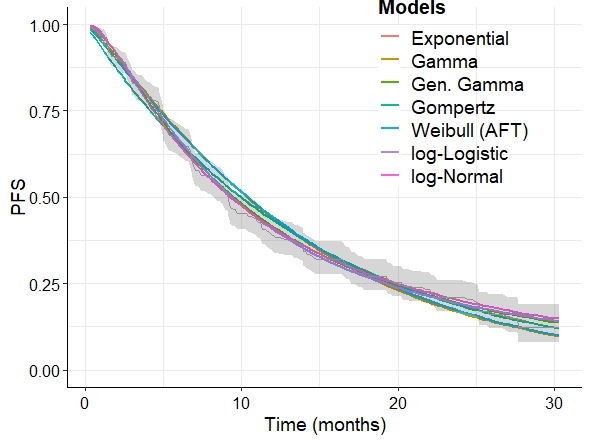

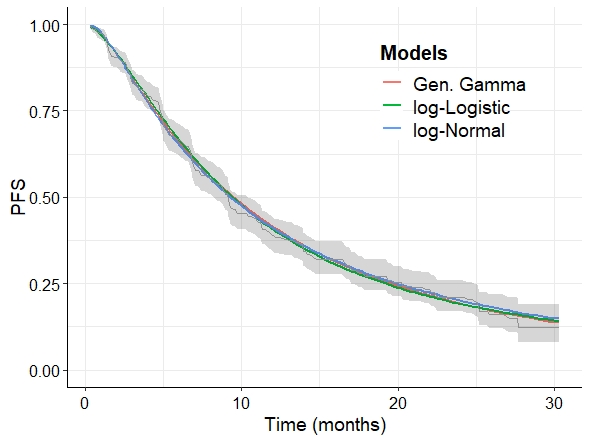


Figure S6. Visual inspection of loglogistic, lognormal, and gengamma distribution for PFS curve in the pembrolizumab group

Figure S5. Visual inspection of seven distributions for PFS curve in the pembrolizumab group

**2.2 PFS curve in the placebo group**

Among the seven distributions, gengamma, loglogistic, and lognormal had the highest statistical fit, considering the values of AIC and BIC (Table S3). The distribution of gengamma was also more consistent with the trend of PFS curve in the placebo group, based on the KEYNOTE-189 trial, according to the visual inspection, which was finally selected into the partitioned survival model (Figure S7-S8).

Table S3. PFS Curve in the Placebo Group

|  | exponential | gamma | gengamma | gompertz | weibull | loglogistic | lognormal |
| --- | --- | --- | --- | --- | --- | --- | --- |
| AIC | 1110.109 | 1091.454 | 1088.212 | 1107.504 | 1096.230 | 1091.100 | 1088.639 |
| BIC | 1113.437 | 1098.109 | 1098.196 | 1114.159 | 1102.886 | 1097.755 | 1095.295 |


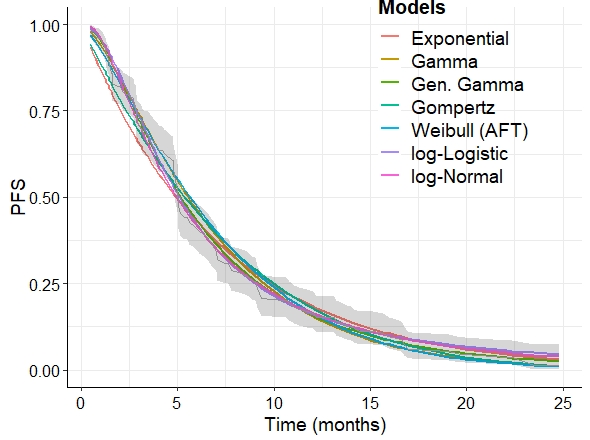

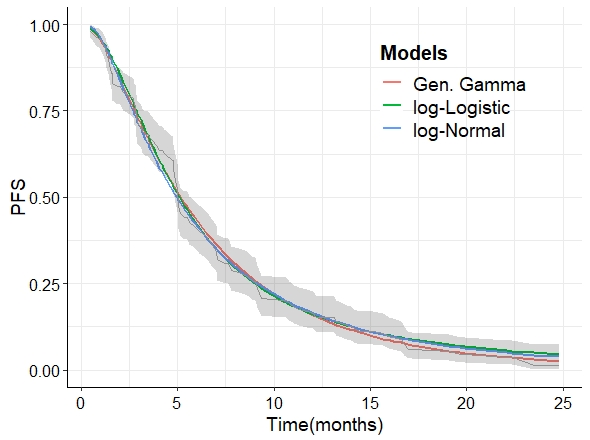


Figure S8. Visual inspection of gengamma, loglogistic and lognormal distribution for PFS curve in the placebo group

Figure S7. Visual inspection of seven distributions for PFS curve in the placebo group

**3. Patients treatment information**

According to KEYNOTE-189 trial, two phases of the medication plans were carried out. Table S4 showed the details of first-line medical treatment and second-line medical treatment for patients in the pembrolizumab and placebo group.

Table S4. Patients’ medical treatment in the pembrolizumab and placebo group

|  | first-line treatment | second-line treatment |
| --- | --- | --- |
| Pembrolizumab Group | 1. pembrolizumab + pemetrexed + carboplatin every 3 weeks for 4 cycles  2. pembrolizumab + pemetrexed for up to 35 cycles  3. pemetrexed maintenance therapy until disease progression | 13.4% of patients were assumed to choose nivolumab.  31.2% of patients were assumed to choose docetaxel. |
| Chemotherapy Group | 1. saline placebo + pemetrexed + carboplatin for 4 cycles  2.saline placebo + pemetrexed maintenance therapy until disease progression | 40.8% patients were crossed over to use pembrolizumab in this study.  13.1% were assumed to choose nivolumab.  5.3% of patients were assumed to choose docetaxel. |
